# Re-evaluating the Concept of Peripheral Hypothyroidism

**DOI:** 10.5812/ijem-173643

**Published:** 2026-09-26

**Authors:** Fereidoun Azizi, Ali Siamak Habibi Moeini

**Affiliations:** 1 Endocrine Research Center, Endocrine Research Center, Research Institute for Endocrine Sciences, Shahid Beheshti University of Medical Sciences Tehran Iran; 2 Endocrine Research Center, Research Institute for Endocrine Sciences, Shahid Beheshti University of Medical Sciences Tehran Iran

**Keywords:** Peripheral Hypothyroidism, Cellular Hypothyroidism, Slow-release T3, Thyromimetics

## Abstract

**Context:**

Isolated peripheral hypothyroidism comprises a group of disorders in which cellular thyroid hormone action is impaired because of defects in membrane transport or intracellular metabolism, despite normal or increased thyroid hormone secretion. Because serum T4, T3, and TSH concentrations often remain within the normal range and sensitive markers of tissue thyroid status are lacking, diagnosis remains challenging. This review examines the causes of isolated peripheral hypothyroidism and recent advances in its management.

**Evidence Acquisition:**

A MEDLINE search covering 2010 - 2025 was conducted using the terms peripheral hypothyroidism, tissue-specific hypothyroidism, and cellular hypothyroidism, supplemented by manual screening of reference lists. After abstract screening, full-text articles were assessed for eligibility, duplicates were removed, and only studies with appropriate designs were included.

**Results:**

The causes can be categorized into four groups: 1) decreased peripheral T3 availability, as observed in patients receiving levothyroxine (LT4) monotherapy who have persistent symptoms associated with low serum T3:T4 ratios despite normal serum T4, T3, and TSH concentrations; 2) peripheral cellular dysfunction, reported in metabolic dysfunction-associated steatotic liver disease (MASLD), skeletal muscle changes during weight regain, and organ-specific conditions involving the heart, lungs, and kidneys, suggesting widespread tissue involvement; 3) altered deiodinase activity, including shifts toward reverse T3 production during cellular dysfunction and consumptive hypothyroidism caused by marked DIO3 overexpression in tumors such as hemangiomas; and 4) thyroid hormone receptor alterations, such as thyroid hormone receptor mutations causing resistance.

**Conclusions:**

Isolated peripheral hypothyroidism comprises a diverse and often underrecognized group of disorders because standard biochemical tests may appear normal. Promising therapies include thyroid hormone combinations, such as levothyroxine plus slow-release T3, and thyromimetics, such as resmetirom for MASLD. This review synthesizes recent findings on cellular hypothyroidism, diagnostic challenges, and emerging multidisciplinary management strategies. Improved biomarkers and multidisciplinary evaluation are still needed to advance its diagnosis and management.

## 1. Context

Thyroid hormones are essential for regulating the metabolism of all cells and organs in the human body. Hypothyroidism, a state of deficient thyroid hormone action, is a common disorder in the general population. Traditionally, hypothyroidism has been classified as primary, central, or peripheral. Primary hypothyroidism is common and is caused mainly by iodine deficiency and Hashimoto thyroiditis. It is characterized by decreased serum thyroid hormone concentrations and an elevated TSH concentration. Central hypothyroidism is a rare disorder caused by pituitary or hypothalamic pathology. Both primary and central hypothyroidism cause peripheral tissue hypothyroidism; however, isolated peripheral hypothyroidism refers to a diverse group of disorders in which defects in cell membrane transport and metabolism reduce the cellular effectiveness of thyroid hormone. In these disorders, thyroid hormone secretion is normal or increased, but thyroid hormone action in peripheral tissues is impaired (1).

Genetic alterations may cause isolated peripheral hypothyroidism by reducing tissue sensitivity to the biological activity of chemically intact hormones, resulting in tissue-specific hypothyroidism (2). Consumptive hypothyroidism is another rare form of peripheral hypothyroidism that occurs when certain tumors, such as infantile hepatic hemangiomas, increase the expression of type 3 iodothyronine deiodinase (DIO3), which then inactivates thyroid hormone (3).

In addition to these rare diseases, isolated peripheral hypothyroidism has been reported in several clinical settings. Specifically, decreased thyroid hormone effectiveness may occur in patients with hypothyroidism who are receiving adequate doses of levothyroxine (4) and in patients with hepatocellular dysfunction (5). This narrative review explores the causes of isolated peripheral hypothyroidism and recent advances in the management of this diverse condition.

## 2. Evidence Acquisition

A comprehensive MEDLINE search was conducted to identify studies investigating peripheral hypothyroidism. The search terms used were "peripheral hypothyroidism," "tissue-specific hypothyroidism," "cellular hypothyroidism," "deiodinase activity," "thyroid hormone receptors," "levothyroxine and thyroid hormone status," "intrahepatic hypothyroidism," "respiratory hypothyroidism," and "renal hypothyroidism." Additional relevant studies were identified by manually reviewing the reference lists of the retrieved articles. The title search yielded more than 2490 results, and 81 publications contained primary data or suitable reviews relevant to the scope of this review.

## 3. Results

Figure 1 illustrates peripheral thyroid hormone transport and metabolism in target tissues. The causes of isolated peripheral hypothyroidism can be categorized into four groups: decreased peripheral availability of T3, peripheral tissue cellular dysfunction, altered deiodinase activity, and thyroid hormone receptor alterations.

**Figure 1.**
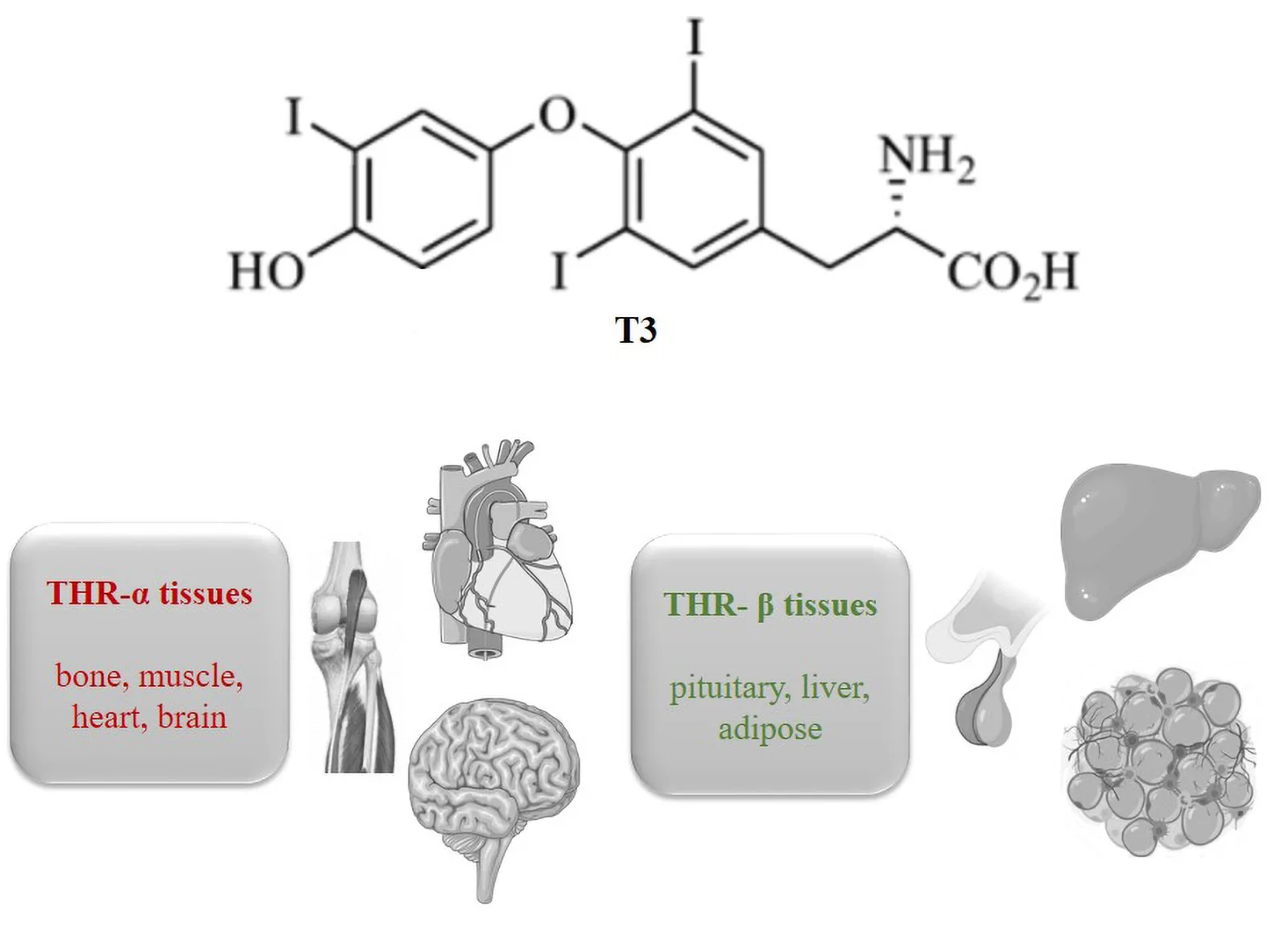
Schematic diagram of thyroid hormone transport and activation in the target tissue, showing alterations leading to isolated peripheral hypothyroidism: 1. Decreased peripheral availability of T3; 2. Cellular dysfunction; 3. Altered activity of deiodinases; 4. Thyroid hormone receptor alteration.

### 3.1. Decreased Peripheral Availability of T3

Patients receiving levothyroxine (LT4) monotherapy may experience peripheral symptoms or other manifestations of hypothyroidism, which have been attributed to a decreased serum T3:T4 ratio despite normal serum T4, T3, and TSH concentrations in most LT4-treated patients (6).

The ability of the human body to generate T3 in patients with hypothyroidism treated with LT4 has been recognized since classic studies demonstrated normal serum T3 levels during LT4 monotherapy (7). Subsequent research showed that when fT4 or the T4 index was elevated, the serum T3:T4 ratio in LT4-treated patients was lower than that in euthyroid controls (8). In thyroidectomized rats, T4 alone failed to normalize plasma and tissue T3 levels, even at the expense of supraphysiologic T4 levels (9).

Saravanan et al. showed that LT4-treated patients with hypothyroidism, including those with normal serum TSH concentrations, had significant deficits on psychological well-being tests compared with normal controls. The authors suggested that substantial psychological morbidity occurred in a considerable proportion of LT4-treated individuals (10). However, in their 2006 report, they found that the General Health Questionnaire correlated with fT4, whereas serum fT3 did not correlate with the General Health Questionnaire or Thyroid Symptom Scores (11). Gullo et al. stated that LT4 monotherapy cannot guarantee euthyroidism in athyreotic patients because these individuals have highly heterogeneous T3 production capacity from orally administered LT4, resulting in an abnormal T3:T4 ratio in approximately one-third of patients (6). Samuels et al. found normal energy expenditure and body composition in LT4-suppressed participants but decreased energy expenditure in LT4-treated euthyroid women, which was associated with lower free T3 concentrations (12). An analysis of National Health and Nutrition Examination Survey data by Peterson et al. revealed lower serum fT3:fT4 ratios in LT4 users, with metabolic differences from controls in 12 of 52 variables. Age, female sex, and serum creatinine were negatively associated with the serum fT3:fT4 ratio, whereas BMI, total cholesterol, and triglycerides were positively associated with it (13). Previous studies found higher BMI and adverse lipid profiles in LT4-treated patients than in euthyroid patients with Graves disease receiving long-term methimazole therapy (4, 14), and a meta-analysis of 99 LT4 monotherapy studies confirmed elevated serum LDL cholesterol and triglyceride concentrations in LT4-treated participants (15). However, the contribution of the T3:T4 ratio to differences in some variables between LT4-treated individuals and the general population remains unclear, although the fT3:fT4 ratio correlates more strongly with metabolic parameters than TSH does in euthyroid individuals (16).

### 3.2. Peripheral Tissue Cellular Dysfunction

A major conceptual challenge is distinguishing isolated peripheral hypothyroidism from nonthyroidal illness. In many patients with nonthyroidal disease, changes occur in the regulation of the hypothalamic-pituitary-thyroid axis and in thyroid hormone transport and metabolism; this condition is known as nonthyroidal illness syndrome (NTIS) (17). Serum T3 concentrations are decreased and reverse T3 (rT3) concentrations are increased, whereas serum T4, FT4, and TSH concentrations remain normal in most patients. However, severely ill patients may have low serum T4 concentrations, which are inversely correlated with mortality (17, 18). The absence of an increase in serum TSH despite a marked decrease in serum T3 and sometimes T4 may be due to reduced sensitivity of TSH secretion to low thyroid hormone concentrations in NTIS (19). Decreased deiodinase 1 (DIO1) activity and increased DIO3 activity explain the low serum T3 and high rT3 concentrations in NTIS, particularly in the liver (20).

The absence of nonthyroidal illness and the absence of reduced serum T3 and increased serum rT3 concentrations may help distinguish isolated peripheral hypothyroidism from NTIS. However, this distinction may be difficult in mild cases.

Over the past two decades, several animal studies have demonstrated isolated peripheral hypothyroidism in various tissues, particularly the liver, heart, muscle, lung, kidney, and brain. Some human studies have also reported peripheral hypothyroidism in conditions with an intact hypothalamic-pituitary-thyroid axis. Isolated tissue hypothyroidism has been reported mainly in hepatic tissue (5) and skeletal muscle (21). Cellular dysfunction alters the expression and activity of deiodinases that govern thyroid hormone activation and inactivation. DIO1 is expressed predominantly in the liver and kidney, whereas DIO2 is found primarily in the pituitary gland and brain, brown adipose tissue, and skeletal muscle (22).

#### 3.2.1. Intrahepatic Hypothyroidism

A potential link between hypothyroidism and metabolic dysfunction-associated steatotic liver disease (MASLD) was proposed in 2003 (23). In NHANES III, a large US population with a median follow-up of 23 years, subclinical hypothyroidism was associated with higher all-cause and cardiovascular mortality in patients with MASLD than in normal controls (24). More recent data suggest that MASLD pathogenesis may be driven in part by intrahepatic hypothyroidism (Figure 2), characterized by reduced thyroid hormone concentrations within hepatocytes despite normal serum thyroid hormone concentrations (25).

**Figure 2.**
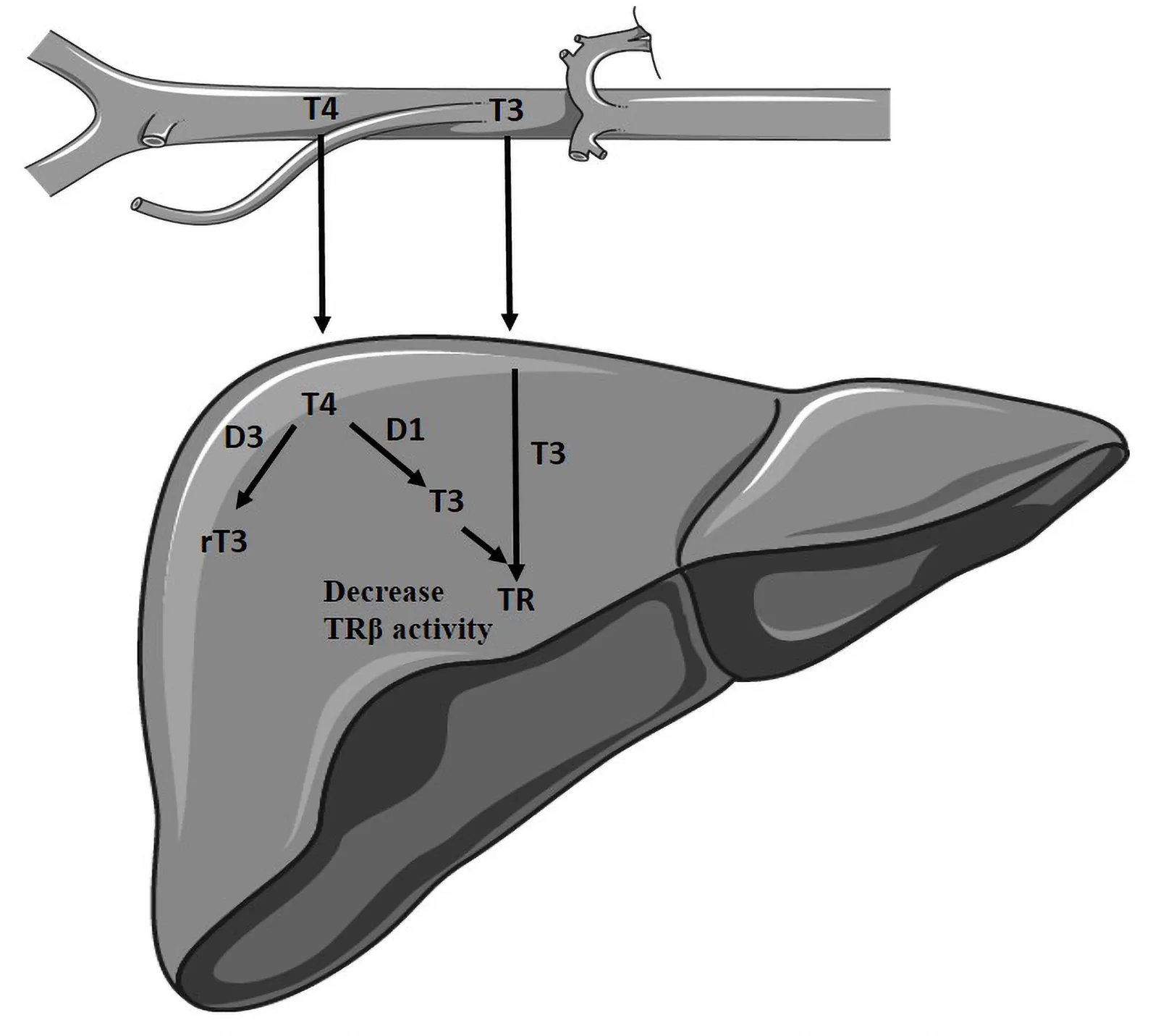
Intrahepatic hypothyroidism in MASLD: Downregulation of deiodinase 1 and upregulation of deiodinase 3 result in decreased TRβ activity.

In the liver, DIO1 is the main deiodinase that converts T4 to T3. During liver injury, there may be a switch from DIO1 to DIO3, which converts T4 to reverse T3 (rT3). This switch is mediated by activation of the hedgehog pathway, an injury-induced signaling cascade triggered by liver injury (26). These enzyme changes reduce the local generation of bioactive T3 and downregulate T3-responsive hepatic gene expression (27), resulting in liver-specific hypothyroidism without changes in serum T4, T3, and TSH concentrations (28).

Reduced activation of thyroid hormone receptor β (THRβ) in hepatocytes promotes hepatic steatosis, impairs mitochondrial biogenesis and mitophagy, increases the generation of reactive oxygen species, and increases hepatic and circulating cholesterol concentrations (29, 30, 31).

#### 3.2.2. Skeletal Muscle Hypothyroidism

Peripheral hypothyroidism in skeletal muscle refers to tissue-specific thyroid hormone resistance or deficiency despite normal serum T4, T3, and TSH concentrations and may be induced by impaired thyroid hormone transport, altered deiodinase activity, or altered thyroid hormone receptor signaling. Skeletal muscle, which accounts for 30 - 40% of body mass (32), is a major thyroid hormone target organ involved in contractility, energy metabolism, and regeneration.

Peripheral hypothyroidism disrupts T3 activity in myocytes through downregulation of DIO2, upregulation of DIO3, dysfunction of monocarboxylate transporter 8 (MCT8), and the accumulation of glycosaminoglycans in type II fibers (33). In sarcopenia, low T3 accelerates protein catabolism and interferes with satellite cell fusion (34). In cachexia, thyroid hormone resistance develops after denervation, and increased atrogin-1, myostatin, and proinflammatory cytokines (IL-6 and TNF-α) together impair thyroid hormone receptor nuclear translocation (35).

An example of skeletal muscle hypothyroidism has been proposed by Dulloo in the context of adaptive thermogenesis during catch-up fat (21). Adaptive thermogenesis during weight loss primarily arises from central suppression of the sympathetic nervous system and the hypothalamic-pituitary-thyroid axis (36, 37). During refeeding, rapid increases in leptin and insulin restore these central pathways and reestablish control of thermogenesis. However, studies have shown resistance to the actions of the restored systemic neurohormonal network on muscle thermogenesis during weight regain, associated with local alterations in deiodinase enzymes within myocytes (38, 39). DIO2 expression decreases, while DIO3 expression and activity increase, resulting in diminished net muscle T3 neogenesis from FT4 and increased rT3 production (32). Diminished net T3 neogenesis may be accompanied by several skeletal muscle metabolic and contractile features observed in clinical hypothyroidism (40, 41).

These changes may be accompanied by reduced muscle strength, increased muscle insulin resistance, impaired myogenesis and muscle regeneration, and disproportionate fat accumulation, thereby increasing the risk of sarcopenic obesity (35, 42).

#### 3.2.3. Peripheral Hypothyroidism in Ischemic Heart Disease

Peripheral hypothyroidism describes cardiomyocyte-specific thyroid hormone deficiency or resistance despite normal serum hormone concentrations, primarily due to upregulated DIO3, which inactivates thyroid hormone and shifts local metabolism toward rT3 production. In ischemic heart disease, including stable angina, acute coronary syndrome, and myocardial infarction, this local hypothyroid state exacerbates injury, impairs remodeling, and worsens prognosis (43). Hypoxia upregulates DIO3 within hours, creating T3-deficient zones that inactivate thyroid hormone and attenuate local signaling (44).

#### 3.2.4. Peripheral Hypothyroidism in Lung Injury

Peripheral lung hypothyroidism denotes tissue-specific thyroid hormone resistance or deficiency in pulmonary cells despite normal circulating thyroid hormone concentrations. Lung injury upregulates DIO3 and downregulates DIO2 and THRβ, shifting local metabolism toward rT3 production (45). This pattern is observed in acute respiratory distress syndrome, hyperoxia-induced bronchopulmonary dysplasia, and lipopolysaccharide-induced inflammation and may aggravate chronic conditions such as chronic obstructive pulmonary disease or asthma. Cellular hypothyroidism in the lung worsens alveolar damage, impairs repair, and increases mortality (46).

#### 3.2.5. Peripheral Hypothyroidism in Chronic Kidney Disease

Peripheral hypothyroidism denotes thyroid hormone deficiency or resistance at the renal cellular level despite normal serum thyroid hormone concentrations and is driven by upregulated DIO3 and downregulated DIO1 and THRβ expression. Chronic kidney disease (CKD), affecting 10 - 15% of the global population (47), has a bidirectional interaction with thyroid function. Hypothyroidism accelerates CKD progression, whereas CKD itself induces low T3 syndrome in 50 - 80% of advanced cases (48, 49).

### 3.3. Altered Deiodinase Activity

The prohormone T4 is produced by the thyroid gland and must be activated to T3 by deiodination. Approximately 80% of T3 produced each day results from extrathyroidal deiodination by DIO1 and DIO2. Both T4 and T3 are also converted to inactive forms through deiodination by DIO1 or DIO3. Deiodinases therefore play an important role in regulating plasma and tissue T3 concentrations (22, 50).

Deiodinase alterations are well documented in human disorders. Extrathyroidal conversion of T4 to T3 is altered in patients with severe nonthyroidal illnesses, various cancers, ischemic heart disease, lung injury, and MASLD. Although nonthyroidal illnesses often alter serum thyroid hormone and TSH concentrations, other conditions may produce isolated cellular hypothyroidism and warrant further clinical investigation.

A distinct clinical condition termed consumptive hypothyroidism has been described primarily in children with hemangiomas. Very high levels of DIO3 may be expressed, resulting in enhanced deiodination of T4 to rT3 (51). The rate of T4 degradation by DIO3 expressed in these tumors can approach the synthetic capacity of even a TSH-stimulated normal human thyroid.

Data on the association between DIO2 polymorphisms and the response to hypothyroidism treatment are limited. One study found that individuals harboring the Thr92Ala DIO2 polymorphism had a poorer psychological response to LT4 therapy than individuals with other genotypes, which may partly explain the lack of benefit from LT4 therapy (52). However, other studies reported no effect of this polymorphism on quality of life or cognition (53), and one study found a detrimental effect of a DIO1 polymorphism (54).

### 3.4. Thyroid Hormone Receptor Alterations

Thyroid hormone exerts nuclear effects through thyroid hormone receptors (THRs), including α and β receptors. T3 binds to THRs with 10- to 15-fold greater affinity than T4. THRα is predominant in the brain, heart, skeletal muscle, and bone, whereas THRβ is expressed most strongly in the liver, kidney, pituitary, and adipose tissue (Figure 3). Mutations in THRs may cause resistance to thyroid hormone action (55). Patients with thyroid hormone resistance caused by THRβ mutations are characterized by goiter, elevated serum T4 concentrations, and normal or slightly elevated TSH concentrations that respond to TRH (2); therefore, they typically do not meet the criteria for isolated peripheral hypothyroidism.

**Figure 3.**
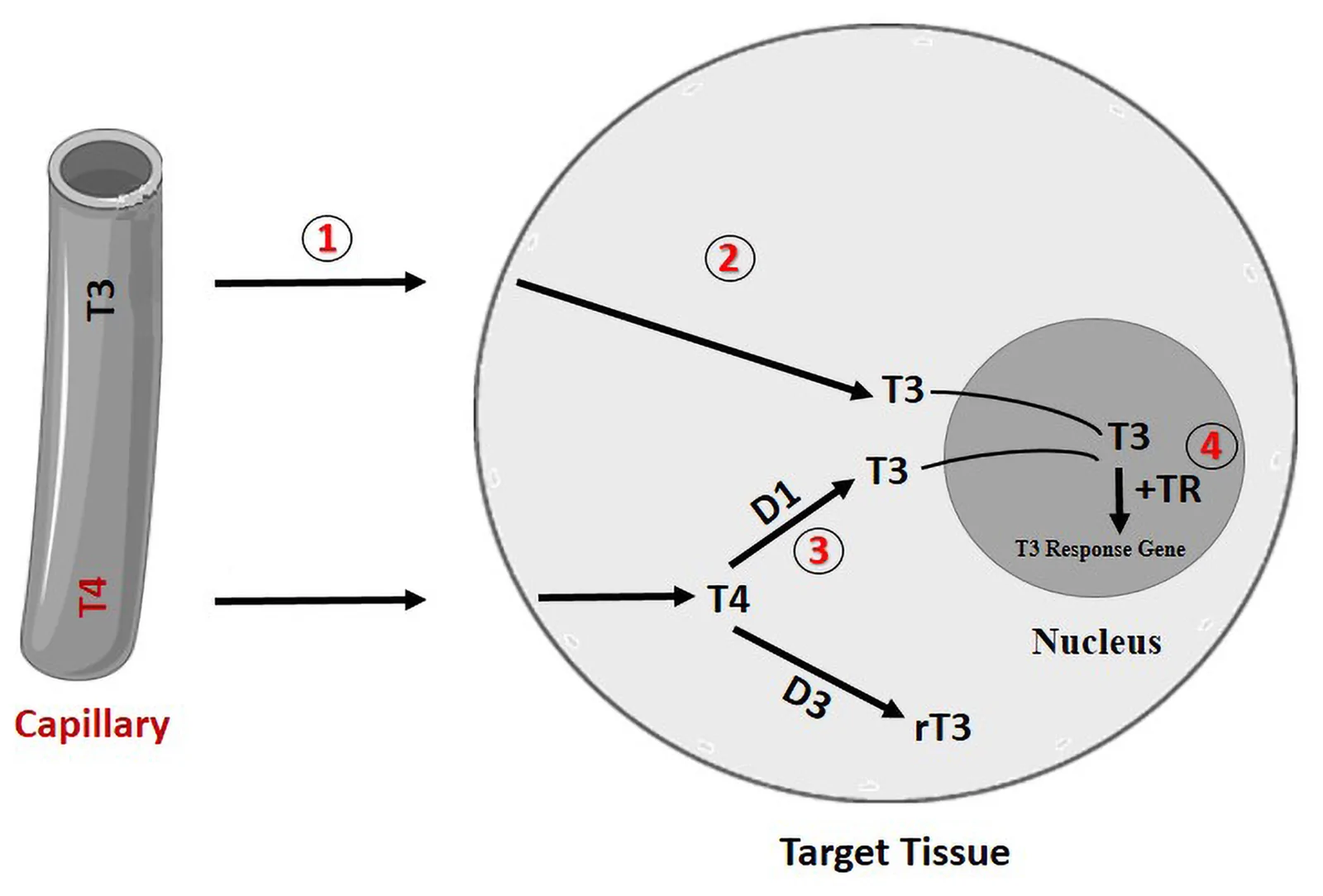
Exertion of T3 through thyroid α and β receptors.

### 3.5. Emerging Therapies

To deliver an adequate amount of T3 to thyroid hormone receptors, treatment with thyroid hormone preparations and thyromimetics has been investigated. In specific clinical settings, GLP-1 receptor agonists may also yield promising results.

#### 3.5.1. Thyroid Hormones

##### 3.5.1.1. Levothyroxine

In conditions with normal thyroid hormone secretion and peripheral hypothyroidism, excess T4 availability to cells cannot deliver more T3 to receptors without inducing a hyperthyroid state. Indeed, levothyroxine treatment of patients with MASLD has been ineffective in preventing the progression of fibrosis and cirrhosis (56).

##### 3.5.1.2. Combination LT4 and LT3 Therapy

Clinical trials of combination therapy with LT4 and LT3 have been reported frequently (57, 58). This combination increases serum thyroid hormone concentrations and the FT3/FT4 ratio, which may be higher than, equal to, or lower than the ratio in healthy individuals (59). However, despite a normal FT3/FT4 ratio, combination therapy was not superior to LT4 monotherapy with respect to quality of life, mood, fatigue, anxiety, or depression (58, 59). Therefore, the 2021 ATA/ETA joint consensus concluded that combination therapy has not shown consistent benefit and is not routinely recommended (60).

##### 3.5.1.3. Slow-Release T3 Formulations

One study showed that LT4 combined with slow-release T3 (SRT3) improved serum T4/T3 and T3 levels and the T3/T4 ratio compared with LT4 monotherapy; however, the T3/T4 ratio did not reach levels comparable with those in euthyroid individuals (61). More recent studies using SRT3 plus LT4 have reported adequate pharmacodynamic and pharmacokinetic properties, with minimal serum T3 excursions over 24 hours and a T3/T4 ratio within normal limits (62, 63). An SRT3/LT4 ratio of 1:5 was found to be optimal for normalizing serum FT4, T3, and TSH concentrations, as well as the T3/T4 ratio. The effects of this combination on symptoms and quality of life and the safety of long-term therapy require further investigation (64). The impact of SRT3 on the management of peripheral hypothyroidism in the liver, muscle, heart, lungs, kidneys, and other tissues also requires further study.

An increased risk of mortality, particularly from cardiovascular causes, has been reported in patients treated for hypothyroidism; this increased risk persists even among well-managed patients with on-target TSH concentrations (65-67). Treatment with T3 preparations to restore euthyroidism in isolated hypothyroid tissues may induce hyperthyroidism in other organs, such as the heart, where THRα receptors are prominent. A recent meta-analysis showed that hypothyroidism treatment containing LT3 was associated with reduced risks of dementia and mortality (68). Further studies may clarify the safety of LT4 plus LT3 combination therapy.

The efficacy of thyroid hormone treatment in patients with NTIS remains controversial. Most randomized studies have not confirmed a beneficial effect of L-T4 or L-T3 treatment in NTIS (69). Treatment with hypothalamic releasing factors such as TRH remains a potential approach (70).

#### 3.5.2. Thyromimetics

Thyromimetics have a molecular structure similar to that of thyroid hormones but differ in their selective receptor activation (71). Resmetirom, the first FDA-approved therapy for MASLD, is an orally administered, liver-targeted thyroid hormone receptor agonist with 83.8 - 90% of the efficacy of T3 in activating THRβ, whereas THRα activation is only 25 - 48.6% (5, 72). By activating hepatocyte THRβ, resmetirom enhances fatty acid oxidation and mitochondrial biogenesis and reduces LDL cholesterol concentrations (73), while minimizing undesirable systemic effects on the heart and bone, which are primarily mediated through THRα (74). Despite the efficacy of resmetirom in treating MASLD and liver fibrosis, only patients with fibrosis stage F1B, F2, or F3 without cirrhosis were studied, and improvement in fibrosis was observed in only 25 - 30% of participants (75). Thus, additional therapies are still needed.

#### 3.5.3. GLP-1 Receptor Agonists

GLP-1 receptor agonists are considered adjunctive therapeutic agents for MASLD. Resolution of MASH without worsening of fibrosis and a reduction in fibrosis by at least one stage without worsening of steatohepatitis were observed during semaglutide treatment. Semaglutide also produced significant weight loss and improved key cardiometabolic measures. Steatohepatitis resolution and fibrosis improvement occurred in 62.9% and 36.8%, respectively, in the semaglutide group, compared with 34.3% and 14.4%, respectively, in the placebo group (76). Concerns regarding the costs of GLP-1 receptor agonists, substantial weight regain after discontinuation, and their efficacy and safety in patients with cirrhosis may limit this treatment approach in MASLD (77, 78).

## 4. Conclusions

### 4.1. Discussion

The absence of readily accessible tissue-specific markers of thyroid hormone action makes it difficult to confirm a diagnosis of isolated peripheral hypothyroidism. This condition may present as generalized tissue-level hypothyroidism or as hypothyroidism limited to one or several tissues. Symptoms and signs related to peripheral organ alterations may predominate, and mild to moderate clinical manifestations of altered thyroid hormone action, when present, may go undetected.

A variety of proposed tissue markers have been used to quantify thyroid hormone action in peripheral tissues, including serum sex hormone-binding globulin, cholesterol, and ferritin concentrations (79). However, none is sufficiently sensitive or specific for peripheral hypothyroidism to be useful in clinical practice. Given the growing recognition of isolated peripheral hypothyroidism across tissues, including the liver, muscle, bone, heart, kidney, and lung, research should prioritize the identification of novel tissue markers of thyroid hormone action, particularly those related to hypothyroidism in these organs.

Circulating metabolites have increasingly been linked to hypothyroidism (80). Multi-omics insights into potential causal associations between plasma metabolites and peripheral hypothyroidism may provide valuable information for understanding the pathogenesis and, potentially, the diagnosis of this condition (81). Simple tests such as ThyPRO-39 may be used as surrogate measures to assess overall and thyroid-specific quality of life in clinical practice.

A major limitation of this review is that a large proportion of the evidence regarding organ-specific mechanisms in the heart, kidney, and lung is derived from rodent models, and direct human evidence may be lacking. In addition, this review does not adequately distinguish maladaptive tissue hypothyroidism from adaptive conservation.

### 4.2. Future Research Directions

Extensive animal studies have provided important biochemical and histological evidence of isolated cellular hypothyroidism and have suggested possible approaches to restoring reduced tissue effects of thyroid hormone. Human findings of isolated peripheral hypothyroidism, once limited to rare intracellular defects, now extend to MASLD, LT4-treated patients with hypothyroidism, skeletal muscle alterations during weight regain, and peripheral hypothyroidism associated with dysfunction in the heart, lung, kidney, and other organs, suggesting broad tissue involvement. Subtle hypothyroidism may be masked by dominant organ pathology, and no sufficiently sensitive or specific, clinically useful markers of peripheral tissue thyroid status currently exist. Therefore, research should be expanded to identify new thyroid tissue markers for routine clinical use.

The discovery and application of such markers may improve the diagnosis and treatment of isolated peripheral hypothyroidism. Thyromimetic drugs and slow-release T3 preparations may be effective and should be investigated for the treatment of conditions accompanied by cellular hypothyroidism. Future thyroid formulations and strategies may include bioidentical compounded sustained-release T3/T4 tailored to patient-specific needs and experimental delivery systems, such as nanoparticle and transdermal delivery, with the aim of tissue-specific targeting.

### 4.3. Overall Conclusions

Isolated peripheral hypothyroidism is a heterogeneous condition in which thyroid hormone action is impaired in target tissues despite normal or near-normal circulating thyroid hormone and TSH concentrations. Evidence of peripheral hypothyroidism has expanded beyond rare genetic disorders to include dysfunction of the liver, skeletal muscle, heart, lung, and kidney, as well as persistent symptoms in some levothyroxine-treated patients. There are currently no sufficiently sensitive or specific biomarkers for directly assessing thyroid hormone action in peripheral tissues in routine clinical practice. Thyroid hormone preparations and thyromimetics have been investigated to deliver adequate T3 to thyroid hormone receptors. In specific clinical settings, GLP-1 receptor agonists may also offer promising results.

## Data Availability

The dataset presented in the study is available on request from the corresponding author during submission or after publication.

## References

[1] Chaker L, Razvi S, Bensenor IM, Azizi F, Pearce EN, Peeters RP (2022). Hypothyroidism. Nature Reviews Disease Primers.

[2] Refetoff S, Bassett JHD, Beck-Peccoz P, Bernal J, Brent G, Chatterjee K (2014). Classification and proposed nomenclature for inherited defects of thyroid hormone action, cell transport, and metabolism. Thyroid.

[3] Maynard MA, Marino-Enriquez A, Fletcher JA, Dorfman DM, Raut CP, Yassa L (2014). Thyroid hormone inactivation in gastrointestinal stromal tumors. N Engl J Med.

[4] Azizi F, Amouzegar A, Tohidi M, Hedayati M, Cheraghi L, Mehrabi Y (2019). Systemic Thyroid Hormone Status in Treated Graves' Disease. Int J Endocrinol Metab.

[5] Kuchay MS, Isaacs S, Misra A (2024). Intrahepatic hypothyroidism in MASLD: Role of liver-specific thyromimetics including resmetirom. Diabetes Metab Syndr.

[6] Gullo D, Latina A, Frasca F, Le Moli R, Pellegriti G, Vigneri R (2011). Levothyroxine monotherapy cannot guarantee euthyroidism in all athyreotic patients. PLoS One.

[7] Braverman LE, Ingbar SH, Sterling K (1970). Conversion of thyroxine (T4) to triiodothyronine (T3) in athyreotic human subjects. J Clin Invest.

[8] Salmon D, Rendell M, Williams J, Smith C, Ross DA, Waud JM (1982). Chemical hyperthyroidism: serum triiodothyronine levels in clinically euthyroid individuals treated with levothyroxine. Arch Intern Med.

[9] Escobar-Morreale HF, Obregón MJ, Escobar del Rey F, Morreale de Escobar G (1995). Replacement therapy for hypothyroidism with thyroxine alone does not ensure euthyroidism in all tissues, as studied in thyroidectomized rats. J Clin Invest.

[10] Saravanan P, Chau WF, Roberts N, Vedhara K, Greenwood R, Dayan CM (2002). Psychological well-being in patients on 'adequate' doses of l-thyroxine: results of a large, controlled community-based questionnaire study. Clin Endocrinol (Oxf).

[11] Saravanan P, Visser TJ, Dayan CM (2006). Psychological well-being correlates with free thyroxine but not free 3,5,3'-triiodothyronine levels in patients on thyroid hormone replacement. J Clin Endocrinol Metab.

[12] Samuels MH, Kolobova I, Smeraglio A, Peters D, Purnell JQ, Schuff KG (2016). Effects of Levothyroxine Replacement or Suppressive Therapy on Energy Expenditure and Body Composition. Thyroid.

[13] Peterson SJ, McAninch EA, Bianco AC (2016). Is a Normal TSH Synonymous With "Euthyroidism" in Levothyroxine Monotherapy?. J Clin Endocrinol Metab.

[14] Azizi F, Yousefi V, Bahrainian A, Sheikholeslami F, Tohidi M, Mehrabi Y (2012). Long-term continuous methimazole or radioiodine treatment for hyperthyroidism. Arch Iran Med.

[15] McAninch EA, Rajan KB, Miller CH, Bianco AC (2018). Systemic Thyroid Hormone Status During Levothyroxine Therapy In Hypothyroidism: A Systematic Review and Meta-Analysis. J Clin Endocrinol Metab.

[16] Park SY, Park SE, Jung SW, Jin HS, Park IB, Ahn SV (2017). Free triiodothyronine/free thyroxine ratio rather than thyrotropin is more associated with metabolic parameters in healthy euthyroid adult subjects. Clinical Endocrinology.

[17] Docter R, Krenning EP, de Jong M, Hennemann G (1993). The sick euthyroid syndrome: changes in thyroid hormone serum parameters and hormone metabolism. Clin Endocrinol (Oxf).

[18] Fliers E, Bianco AC, Langouche L, Boelen A (2015). Thyroid function in critically ill patients. Lancet Diabetes Endocrinol.

[19] Adriaanse R, Romijn JA, Brabant G, Endert E, Wiersinga WM (1993). Pulsatile thyrotropin secretion in nonthyroidal illness. J Clin Endocrinol Metab.

[20] St. Germain DL, Galton VA, Hernandez A (2009). Minireview: Defining the roles of the iodothyronine deiodinases: current concepts and challenges. Endocrinology.

[21] Dulloo AG (2025). Adaptive thermogenesis driving catch-up fat during weight regain: a role for skeletal muscle hypothyroidism and a risk for sarcopenic obesity. Rev Endocr Metab Disord.

[22] Schweizer U, Steegborn C (2015). New insights into the structure and mechanism of iodothyronine deiodinases. J Mol Endocrinol.

[23] Liangpunsakul S, Chalasani N (2003). Is hypothyroidism a risk factor for non-alcoholic steatohepatitis?. J Clin Gastroenterol.

[24] Kim D, Vazquez-Montesino LM, Escober JA, Fernandes CT, Cholankeril G, Loomba R (2020). Low Thyroid Function in Nonalcoholic Fatty Liver Disease Is an Independent Predictor of All-Cause and Cardiovascular Mortality. Am J Gastroenterol.

[25] Kuchay MS, Choudhary NS, Mishra SK (2020). Pathophysiological mechanisms underlying MAFLD. Diabetes Metab Syndr.

[26] Chng CL, Goh GBB, Yen PM (2025). Metabolic and Functional Cross Talk Between the Thyroid and Liver. Thyroid.

[27] Bohinc BN, Michelotti G, Xie G, Pang H, Suzuki A, Guy CD (2014). Repair-related activation of hedgehog signaling in stromal cells promotes intrahepatic hypothyroidism. Endocrinology.

[28] Isaacs S, Isaacs A (2024). Endocrinology for the hepatologist. Current Hepatology Reports.

[29] Abu-Elheiga L, Brinkley WR, Zhong L, Chirala SS, Woldegiorgis G, Wakil SJ (2000). The subcellular localization of acetyl-CoA carboxylase 2. Proceedings of the National Academy of Sciences of the United States of America.

[30] Rufer AC, Thoma R, Hennig M (2009). Structural insight into function and regulation of carnitine palmitoyltransferase. Cellular and molecular life sciences: CMLS.

[31] Bonde Y, Breuer O, Lütjohann D, Sjöberg S, Angelin B, Rudling M (2014). Thyroid hormone reduces PCSK9 and stimulates bile acid synthesis in humans. Journal of Lipid Research.

[32] Salvatore D, Simonides WS, Dentice M, Zavacki AM, Larsen PR (2014). Thyroid hormones and skeletal muscle--new insights and potential implications. Nat Rev Endocrinol.

[33] Lin N, Wang J, Liao W, Wen Y (2020). Hypothyroid myopathy with periodic paralysis as the main symptom: a case report and literature review. Ann Palliat Med.

[34] De Stefano MA, Ambrosio R, Porcelli T, Orlandino G, Salvatore D, Luongo C (2021). Thyroid Hormone Action in Muscle Atrophy. Metabolites.

[35] Bloise FF, Oliveira TS, Cordeiro A, Ortiga-Carvalho TM (2018). Thyroid Hormones Play Role in Sarcopenia and Myopathies. Front Physiol.

[36] Landsberg L (1986). Diet, obesity and hypertension: an hypothesis involving insulin, the sympathetic nervous system, and adaptive thermogenesis. Q J Med.

[37] Rosenbaum M, Goldsmith RL, Haddad F, Baldwin KM, Smiley R, Gallagher D (2018). Triiodothyronine and leptin repletion in humans similarly reverse weight-loss-induced changes in skeletal muscle. Am J Physiol Endocrinol Metab.

[38] Aronne LJ, Hall KD, M. Jakicic J, Leibel RL, Lowe MR, Rosenbaum M (2021). Jakicic J, Leibel RL, Lowe MR, Rosenbaum M, et al. Describing the weight‐reduced state: physiology, behavior, and interventions. Obesity.

[39] Di Munno C, Busiello RA, Calonne J, Salzano AM, Miles-Chan J, Scaloni A (2021). Adaptive Thermogenesis Driving Catch-Up Fat Is Associated With Increased Muscle Type 3 and Decreased Hepatic Type 1 Iodothyronine Deiodinase Activities: A Functional and Proteomic Study. Front Endocrinol (Lausanne).

[40] Wiles CM, Young A, Jones DA, Edwards RHT (1979). Muscle relaxation rate, fibre-type composition and energy turnover in hyper- and hypo-thyroid patients. Clin Sci (Lond).

[41] Marcelino H, Veyrat-Durebex C, Summermatter S, Sarafian D, Miles-Chan J, Arsenijevic D (2013). A role for adipose tissue de novo lipogenesis in glucose homeostasis during catch-up growth: a Randle cycle favoring fat storage. Diabetes.

[42] Ogawa-Wong A, Carmody C, Le K, Marschner RA, Larsen PR, Zavacki AM (2022). Modulation of Deiodinase Types 2 and 3 during Skeletal Muscle Regeneration. Metabolites.

[43] Jafar TH, Rasool R, Unar A, Chanihoon GQ, Mubeen B (2023). A Role of Thyroid Hormones in Acute Myocardial Infarction: An Update. Curr Cardiol Rev.

[44] Sorensen JR, Winther KH, Bonnema SJ, Godballe C, Hegedüs L (2016). Respiratory Manifestations of Hypothyroidism: A Systematic Review. Thyroid.

[45] Vamesu BM, Nicola T, Li R, Hazra S, Matalon S, Kaminski N (2023). Thyroid hormone modulates hyperoxic neonatal lung injury and mitochondrial function. JCI Insight.

[46] Rhee CM (2016). The interaction between thyroid and kidney disease: an overview of the evidence. Curr Opin Endocrinol Diabetes Obes.

[47] Jager KJ, Kovesdy C, Langham R, Rosenberg M, Jha V, Zoccali C (2019). A single number for advocacy and communication-worldwide more than 850 million individuals have kidney diseases. Nephrol Dial Transplant.

[48] Coutinho J, Santos C, Rocha E (2019). Hypothyroidism and chronic kidney disease: an undervalued two-way relationship. Port J Nephrol Hypert.

[49] Arrojo e Drigo R, Bianco AC (2011). Type 2 deiodinase at the crossroads of thyroid hormone action. The international journal of biochemistry & cell biology.

[50] Russo SC, Salas-Lucia F, Bianco AC (2021). Deiodinases and the Metabolic Code for Thyroid Hormone Action. Endocrinology.

[51] Huang SA, Tu HM, Harney JW, Venihaki M, Butte AJ, Kozakewich HPW (2000). Severe hypothyroidism caused by type 3 iodothyronine deiodinase in infantile hemangiomas. N Engl J Med.

[52] Panicker V, Saravanan P, Vaidya B, Evans J, Hattersley AT, Frayling TM (2009). Common variation in the DIO2 gene predicts baseline psychological well-being and response to combination thyroxine plus triiodothyronine therapy in hypothyroid patients. J Clin Endocrinol Metab.

[53] Wouters HJCM, van Loon HCM, van der Klauw MM, Elderson MF, Slagter SN, Kobold AM (2017). No Effect of the Thr92Ala Polymorphism of Deiodinase-2 on Thyroid Hormone Parameters, Health-Related Quality of Life, and Cognitive Functioning in a Large Population-Based Cohort Study. Thyroid.

[54] Young Cho Y, Jeong Kim H, Won Jang H, Hyuk Kim T, Ki CS, Wook Kim S (2017). The relationship of 19 functional polymorphisms in iodothyronine deiodinase and psychological well-being in hypothyroid patients. Endocrine.

[55] Yen PM (2015). Classical nuclear hormone receptor activity as a mediator of complex biological responses: a look at health and disease. Best Pract Res Clin Endocrinol Metab.

[56] Rao AK, Rockey DC (2025). Impact of Levothyroxine on Progression to and Complications of Cirrhosis in MASH. Dig Dis Sci.

[57] Celi FS, Zemskova M, Linderman JD, Smith S, Drinkard B, Sachdev V (2011). Metabolic effects of liothyronine therapy in hypothyroidism: a randomized, double-blind, crossover trial of liothyronine versus levothyroxine. J Clin Endocrinol Metab.

[58] Grozinsky-Glasberg S, Fraser A, Nahshoni E, Weizman A, Leibovici L (2006). Thyroxine-triiodothyronine combination therapy versus thyroxine monotherapy for clinical hypothyroidism: meta-analysis of randomized controlled trials. J Clin Endocrinol Metab.

[59] Escobar-Morreale HF, Botella-Carretero JI, del Rey FE, de Escobar GM (2005). REVIEW: Treatment of hypothyroidism with combinations of levothyroxine plus liothyronine. J Clin Endocrinol Metab.

[60] Jonklaas J, Bianco AC, Cappola AR, Celi FS, Fliers E, Heuer H (2021). Evidence-Based Use of Levothyroxine/Liothyronine Combinations in Treating Hypothyroidism: A Consensus Document. Thyroid.

[61] Hennemann G, Docter R, Visser TJ, Postema PT, Krenning EP (2004). Thyroxine plus low-dose, slow-release triiodothyronine replacement in hypothyroidism: proof of principle. Thyroid.

[62] Mehran L, Amouzegar A, Foroutan SM, Masoumi S, Tohidi M, Abdi H (2023). Pharmacodynamic and pharmacokinetic properties of the combined preparation of levothyroxine plus sustained- release liothyronine; a randomized controlled clinical trial. BMC Endocr Disord.

[63] Azizi F, Moeini AS, Mehran L, Masoumi S, Abdi H, Foroutan SM (2025). Treatment of hypothyroidism with the combination of levothyroxine and slow-release triiodothyronine: a randomized clinical trial. J Clin Transl Endocrinol.

[64] Azizi F, Amouzegar A, Abdi H, Tohidi M, Masoumi S, Khalili D (2025). Treatment of hypothyroidism with levothyroxine plus slow-release liothyronine: a study protocol for a randomized controlled double-blinded clinical trial. Trials.

[65] Sohn SY, Seo GH, Chung JH (2021). Risk of All-Cause Mortality in Levothyroxine-Treated Hypothyroid Patients: A Nationwide Korean Cohort Study. Front Endocrinol (Lausanne).

[66] Tsai TY, Tu YK, Munir KM, Lin SM, Chang RHE, Kao SL (2020). Association of Hypothyroidism and Mortality in the Elderly Population: A Systematic Review and Meta-Analysis. J Clin Endocrinol Metab.

[67] Akirov A, Shochat T, Shechvitz A, Shimon I, Diker-Cohen T, Robenshtok E (2017). Pre-admission TSH levels predict long-term mortality in adults treated for hypothyroidism. Endocrine.

[68] Beltrão FEDL, Carvalhal G, Meneghini V, Matos DAR, Beltrão DCDA, Andrade BARM (2026). Treatment of Hypothyroidism That Contains Liothyronine is Associated With Reduced Risk of Dementia and Mortality. The Journal of Clinical Endocrinology & Metabolism.

[69] Kaptein EM, Sanchez A, Beale E, Chan LS (2010). Clinical review: Thyroid hormone therapy for postoperative nonthyroidal illnesses: a systematic review and synthesis. J Clin Endocrinol Metab.

[70] Van den Berghe G, Baxter RC, Weekers F, Wouters P, Bowers CY, Iranmanesh A (2002). The combined administration of GH-releasing peptide-2 (GHRP-2), TRH and GnRH to men with prolonged critical illness evokes superior endocrine and metabolic effects compared to treatment with GHRP-2 alone. Clinical Endocrinology.

[71] Ratziu V, Scanlan TS, Bruinstroop E (2025). Thyroid hormone receptor-β analogues for the treatment of metabolic dysfunction-associated steatohepatitis (MASH). Journal of hepatology.

[72] U.s (2024). Food and Drug Administration.

[73] Abbasi L, Irfan Q, Zaidi SMM, Jawed I, Malik A, Kaleem S (2025). Safety and efficacy of resmetirom in metabolic dysfunction-associated steatohepatitis (MASH): a systematic review and meta-analysis. Eur J Gastroenterol Hepatol.

[74] Lin J, Green V, Sohal A, Roytman M (2025). A Perfect MASH Comparing Resmetirom and GLP-1 Agonists for Metabolic-Associated Steatohepatitis: What Does the Current Evidence Suggest?. J Clin Gastroenterol.

[75] Harrison SA, Bedossa P, Guy CD, Schattenberg JM, Loomba R, Taub R (2024). A Phase 3, Randomized, Controlled Trial of Resmetirom in NASH with Liver Fibrosis. New England Journal of Medicine.

[76] Sanyal AJ, Newsome PN, Kliers I, Østergaard LH, Long MT, Kjær MS (2025). Phase 3 Trial of Semaglutide in Metabolic Dysfunction-Associated Steatohepatitis. New England Journal of Medicine.

[77] Xiao Y, Meng J, Gao S (2024). Tirzepatide for Maintenance of Weight Reduction in Adults With Obesity. Jama.

[78] Simon TG (2025). Semaglutide for Metabolic Dysfunction-Associated Steatohepatitis. New England Journal of Medicine.

[79] Sarne DH, Refetoff S, Rosenfield RL, Farriaux JP (1988). Sex hormone-binding globulin in the diagnosis of peripheral tissue resistance to thyroid hormone: the value of changes after short term triiodothyronine administration. J Clin Endocrinol Metab.

[80] Liu X, Yuan J, Liu S, Tang M, Meng X, Wang X (2023). Investigating causal associations among gut microbiota, metabolites and autoimmune hypothyroidism: a univariable and multivariable Mendelian randomization study. Front Immunol.

[81] Zheng S, Liu L, Liang K, Yan J, Meng D, Liu Z (2024). Multi-omics insight into the metabolic and cellular characteristics in the pathogenesis of hypothyroidism. Communications Biology.

